# Inter-study reproducibility of interleaved spiral phase velocity mapping of renal artery haemodynamics

**DOI:** 10.1186/s12968-014-0105-x

**Published:** 2015-02-04

**Authors:** Jennifer Keegan, Hitesh C Patel, Robin M Simpson, Raad H Mohiaddin, David N Firmin

**Affiliations:** Cardiovascular Magnetic Resonance, Royal Brompton Hospital, Sydney Street, London, SW3 6NP UK; Radiological Physics, University of Freiburg, Freiburg, Germany; National Heart and Lung Institute, Imperial College London, London, UK

**Keywords:** Spiral, Phase velocity mapping, Renal blood flow, Resistive index, Pulsatility index, Reproducibility

## Abstract

**Background:**

Qualitative and quantitative assessment of renal blood flow is valuable in the evaluation of patients with renal and renovascular diseases as well as in patients with heart failure. The temporal pattern of renal flow velocity through the cardiac cycle provides important information about renal haemodynamics. High temporal resolution interleaved spiral phase velocity mapping could potentially be used to study temporal patterns of flow and measure resistive and pulsatility indices which are measures of downstream resistance.

**Methods:**

A retrospectively gated breath-hold spiral phase velocity mapping sequence (TR 19 ms) was developed at 3 Tesla. Phase velocity maps were acquired in the proximal right and left arteries of 10 healthy subjects in each of two separate scanning sessions. Each acquisition was analysed by two independent observers who calculated the resistive index (RI), the pulsatility index (PI), the mean flow velocity and the renal artery blood flow (RABF). Inter-study and inter-observer reproducibility of each variable was determined as the mean +/− standard deviation of the differences between paired values. The effect of background phase errors on each parameter was investigated.

**Results:**

RI, PI, mean velocity and RABF per kidney were 0.71+/− 0.06, 1.47 +/− 0.29, 253.5 +/− 65.2 mm/s and 413 +/− 122 ml/min respectively. The inter-study reproducibilities were: RI −0.00 +/− 0.04 , PI −0.03 +/− 0.17, mean velocity −6.7 +/− 31.1 mm/s and RABF per kidney 17.9 +/− 44.8 ml/min. The effect of background phase errors was negligible (<2% for each parameter).

**Conclusions:**

High temporal resolution breath-hold spiral phase velocity mapping allows reproducible assessment of renal pulsatility indices and RABF.

## Background

Qualitative and quantitative assessment of renal blood flow is valuable in the evaluation of patients with renal and renovascular diseases as well as in patients with heart failure. Abnormalities in renal perfusion resistive index (RI) and pulsatility index (PI) have been noted in many conditions including those affecting the kidneys (e.g. renal artery stenosis) [1] and those primarily affecting other organs with secondary renal insult (e.g., sepsis or liver failure) [2]. The ability to qualitatively and quantitatively measure renal function and blood flow is important in managing patients afflicted with these various diseases.

Recently there has been much interest in the role of renal sympathetic denervation (RSD), a novel percutaneous transcatheter technique for the treatment of patients with hypertension and heart failure [3]. This procedure aims to interrupt the efferent and afferent sympathetic nerves at the renal level and is believed to exert its beneficial effects by improving renal perfusion and renal artery compliance amongst others. Invasive Doppler flow-wire studies in pigs have shown that RSD induces favourable increases in renal artery peak velocity and renal artery blood flow (RABF) coupled with an advantageous decrease in resistive index (RI) [4]. However, there is a limited amount of data in humans on the acute and chronic effects of RSD. Using an invasive approach to study this would be unfeasible in large numbers. The most commonly used non-invasive technique for assessing pulsatility indices is pulse wave Doppler ultrasound although this has both patient and operator dependent limitations. Furthermore, ultrasound is poor at defining renal artery anatomy including visualising accessory vessels and atheroma, which is pivotal before considering a patient for RSD. MR can not only accurately define renal artery anatomy but can also provide haemodynamic data.

While there have been no reports to date on the MR assessment of pulsatility indices in the renal vessels, the analysis of temporal flow velocity patterns in other vessels is an active area of research interest [5,6]. In particular, pulsatility has been measured in the peripheral arteries [7-9], the cerebral arteries [10,11], the ophthalmic arteries [12], the carotid arteries [10,13] as well as in the major vessels [14]. MR assessment of renal artery haemodynamics, however, presents very specific challenges. In particular, their diameters are small relative to the great veins and arteries and they undergo significant motion with the respiratory cycle. In addition, although the kidneys account for less than 1% of body mass, they receive a disproportionately large proportion (20 – 25%) of basal cardiac output [15].

Phase velocity mapping for the assessment of RABF has been generally performed using a segmented gradient echo sequence [16-24] although more recently, segmented field-echo echo-planar [25,26] and spiral [21] techniques have also been implemented. While a rapid non-ECG gated technique has been developed [27-29] to allow assessment of renal artery blood flow (in L/min), ECG-gated acquisitions enable the investigation of instantaneous flow velocities throughout the cardiac cycle and therefore, have the potential to allow the calculation of RI and PI. Respiratory gating may be used to acquire data during free-breathing [21,22,24] but acquisition durations can be long and unpredictable and the majority of studies have instead been performed with breath-hold acquisitions of 16 – 30 s duration [21,22,25,26]. Breath-holding limits the temporal resolution achievable with the number of phases being acquired per cardiac cycle in these studies being 6 [21], 8 [22], 24 [25] and 12 – 24 [26]. This temporal resolution is inferior to that of pulse wave Doppler ultrasound which typically samples at a minimum of 50 Hz (which equates to 50 phases per cardiac cycle for a heart rate of 60 beats per minute). The reproducibility of MR assessment of RABF has been assessed in a number of studies [22-24,26] with varying degrees of success. However, none of these previous studies have assessed clinically important parameters of pulsatility (RI and PI) as they have lacked the high temporal resolution necessary to accurately sample the RABF time curve.

Interleaved spiral phase velocity mapping has the advantage of efficient k-space coverage and RABF values in healthy volunteers using this technique at 1.5 Tesla (temporal resolution of 55 ms, spatial resolution 1.1 mm × 1.1 mm) are in good agreement with measurements made with PAH-clearance haematocrit [21]. The aim of this work is to develop a breath-hold high temporal resolution spiral phase velocity mapping technique at 3 Tesla for the assessment of the temporal flow patterns in the renal arteries in healthy volunteers. Pulsatility indices, RI and PI, will be determined and their inter-observer and inter-study reproducibility assessed. To our knowledge, this is the first time that MR has been used to measure these parameters. Inter-observer and inter-study reproducibility of RABF using this technique are also assessed.

## Methods

An interleaved spiral phase velocity sequence was developed on a 3 Tesla Magnetom Skyra MR scanner (Siemens AG Healthcare Sector, Germany) equipped with an 18-element cardiac coil and a 48-element spine coil. A 1–1 water excitation (duration = 3 ms) was implemented which eliminated off-resonance blurring of fat and full k-space coverage was achieved in 8 spiral interleaves of 11.75 ms duration. The spiral trajectory was slew rate limited (to 150 T/m/s) until a gradient amplitude of 32 mT/m was reached, and thereafter it was gradient amplitude limited (32 mT/m). The trajectory consisted of 4700 points sampled at 2.5 us intervals. The interleaves were incremented linearly. Phase map subtraction of datasets with symmetric bi-polar velocity encoding gradients resulted in through-plane velocity maps where a phase shift of +/− 180° represented a flow velocity +/− 150 cm/s. Following a single dummy cycle, these velocity encoded datasets were acquired in alternating cardiac cycles in an end-expiratory breath-hold of 17 cardiac cycles duration. The sequence TE was 5.2 ms, and the TR was 19 ms. Data were reconstructed online following gridding onto a 256 × 256 matrix using a standard gridding algorithm [30]. The number of coil elements used was limited to 6 from the anteriorly-positioned cardiac coil and 6 from the posterior spine coil. This reduced the reconstruction time and also minimised wrap. The slice thickness was 8 mm, the spatial resolution 1.4 × 1.4 mm (reconstructed to 0.7 × 0.7 mm through zero-filling) and the repeat time (acquired temporal resolution) 19 ms. Retrospective ECG gating allowed full coverage of the entire cardiac cycle in 50 cine frames, the reconstructed temporal resolution depending on the subjects’ heart rates. Retrospective ECG gating results in more complete sampling of the temporal flow wave form than prospective gating (as there is no gating dead time) and, provided that beat to beat variations in the R-R interval length during the acquisition are small, should therefore result in a more accurate measure of mean flow velocity.

The study was approved by the National Research Ethics Service and all subjects gave written informed consent. Cross-sectional proximal left and right renal artery phase velocity maps, were acquired in 10 healthy volunteers (aged 24–37 years, 8 male). The subjects were scanned supine with the origin of the renal arteries positioned at iso-centre to reduce background phase errors. The image planes were determined following diastolic scout acquisitions with an ECG-gated breath-hold segmented gradient echo sequence (TE/TR: 3.3 ms/7 ms, acquired resolution: 1 mm × 1 mm × 4 mm, acquisition window 110 ms) and in each case, were positioned in a straight section of artery, 1 – 2 cm from its origin. Sensitivity to off-resonance was minimised by localised second-order shimming and frequency adjustment based on the signal from a user-defined region of interest positioned over the descending aorta and renal arteries. Left and right renal phase velocity maps were acquired twice, once in each of two separate scanning sessions with the volunteer leaving the scanner between sessions. The typical time between scanning sessions was 30 minutes.

For each artery in each scanning session, background phase errors were determined for each acquired dataset by scanning a large homogeneous stationary phantom using the same sequence parameters and the same slice positions as in the volunteer study [31]. A simulated ECG with the same heart-rate as the subject was used to trigger the acquisitions.

### Analysis

The left and right vessels in each scanning session were analysed independently by two observers who each had more had more than 5 years experience with CMR imaging and who trained both individually and together to draw regions of interest around the renal arteries. For each vessel in each scanning session, the cross-sectional area was manually defined on the cross-sectional segmented gradient echo image after adjusting the window width and level to 80% and 60% of the maximum pixel value in the vessel respectively. This region of interest was copied to all magnitude images in the corresponding spiral acquisition. For each cine frame, the position of the region was adjusted, as required, and then copied to the corresponding velocity map. The size of the region was not adjusted. The mean velocity in this region of interest was plotted through the cardiac cycle and the mean flow velocity over time and the absolute flow determined. Measures of pulsatility, RI and PI, were determined as:

$$ \begin{array}{cc}\hfill \mathrm{R}\mathrm{I}=\left(\mathrm{P}\mathrm{S}\mathrm{V}\hbox{--} \mathrm{M}\mathrm{D}\mathrm{V}\right)/\mathrm{P}\mathrm{S}\mathrm{V}\hfill & \hfill \mathrm{PI}=\left(\mathrm{P}\mathrm{S}\mathrm{V}\hbox{--} \mathrm{M}\mathrm{D}\mathrm{V}\right)/\mathrm{M}\mathrm{V}\hfill \end{array} $$

where: PSV = peak systolic velocity, MDV = minimum diastolic velocity and MV = mean velocity through the cardiac cycle.

To assess background phase errors, all datasets were processed three times: (i) with no background phase correction, (ii) following fitting of a background phase map to user defined stationary points in the inter-vertebral disks [27] and (iii) using the velocity maps from the subject-specific large stationary phantom acquisitions [31]. While the latter requires time-consuming additional data acquisitions, it is currently regarded as the most accurate method of background correction. As data were acquired with retrospective ECG-gating, for both correction methods, the average background phase throughout the entire cardiac cycle was subtracted from the uncorrected data. After checking the data for normality using the Shapiro-Wilk test, a repeated measures analysis of variance was used to compare quantitative parameters derived from the flow velocity curves (RI, PI, mean velocity and flow) with the three background correction techniques (none, background fit and stationary phantom). If statistically significant, paired t-tests (with Bonferonni correction for multiple testing) were performed to assess differences between the techniques.

The inter-observer reproducibility of cross-sectional area, RI, PI, mean velocity and flow in the initial scanning session were determined as the mean +/− standard deviation of the signed differences between the two observers. For each observer, the inter-study reproducibilities of these variables were determined as the mean +/− standard deviation of the signed differences between the initial and the repeated scans. For each parameter and each observer, intraclass correlation coefficients (ICCs) (using a two-way mixed effects model with average measures) and within-subject coefficients of variation (CVs) were also calculated. The CV was calculated as follows [32]:

$$ \mathrm{C}\mathrm{V}\left(\%\right)=100\%\times \left(\mathrm{within}-\mathrm{subject}\ \mathrm{standard}\ \mathrm{deviation}/\mathrm{mean}\right) $$

where:

- within-subject standard deviation is √(∑((meas_1_ – meas_2_)^2^)/2n)
- meas_1_ and meas_2_ are the paired readings in each artery
- n is the number of paired readings
- mean = the average of all measurements in all arteries

All analyses were performed using IBM SPSS Statistics 19 Package.

## Results

**Figure 1:**
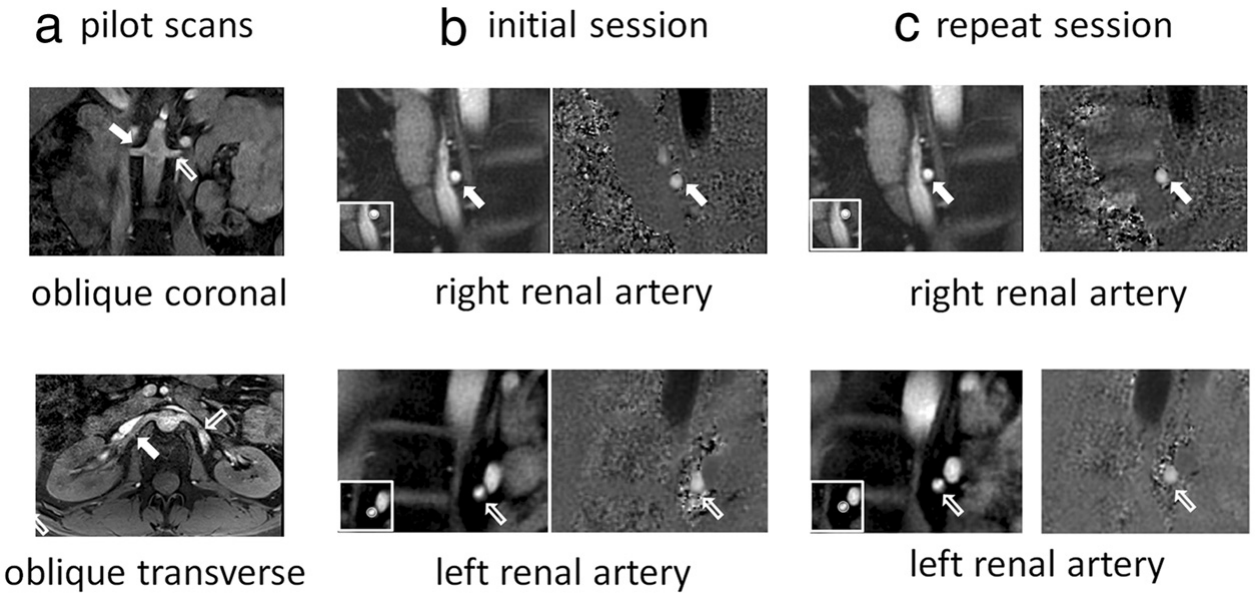
**Oblique coronal (top) and oblique transverse (bottom) pilot images showing the proximal paths of the left and right renal arteries in an example subject (a) together with through-plane systolic magnitude images and velocity maps from both scanning sessions (initial (b) and repeat (c)).** The renal artery regions of interest are shown in inserts images to the bottom left of the magnitude images. (open arrow = left renal artery, solid arrow = right renal artery).

Left and right renal artery data were acquired in all 10 subjects in 2 scanning sessions (40 acquisitions in total). The left renal acquisition in the repeat scanning session of one subject was eliminated due to ECG mis-triggerring. The mean RR interval durations in the initial and repeat scanning sessions were 857 +/− 101 ms and 851 +/− 76 ms respectively (paired t-test: P = 0.67). Figure 1 shows oblique coronal and oblique transverse pilot images showing the proximal paths of the left and right renal arteries in an example subject (a) together with through-plane systolic magnitude images and velocity maps from both scanning sessions (initial (b) and repeat (c)). RI, PI, mean velocity and RABF per kidney (observer 1, initial scan) were 0.71+/− 0.06, 1.47 +/− 0.29, 253.5 +/− 65.2 mm/s and 413 +/− 122 ml/min, respectively.

Table 1 shows the mean (+/− standard deviation) back ground phase velocity together with the RI, PI, mean velocity and RABF per kidney for the acquisitions in the initial scanning session when analysed without correction for background phase together with the same parameters when analysed with the background fit correction and with the stationary phantom correction. The background velocity error is small, being 2.9 +/− 3.3 mm/s for the stationary phantom and 4.6 +/− 7.4 mm/s for the background fit. Repeated measures analysis of variance showed significant differences in measurements of RI, PI, mean velocity and RABF between the techniques. Subsequent paired t-testing showed very small but statistically significant differences between the stationary phantom corrected measurements and those derived without background correction, the mean difference (as a percentage of the uncorrected values) being 0.0%, 1.3%, 1.1% and 1.0% for RI, PI, mean velocity and RABF respectively. There were similarly small differences between the background fit corrected data and the uncorrected data: 1.4%, 2.0%, 1.8% and 1.7% for RI, PI, mean velocity and RABF respectively. As the differences are so small and clinically unimportant, all subsequent results are presented without background correction.

**Table 1:** **Background velocity, flow, mean flow velocity, RI and PI for the renal arteries analysed with different methods of background phase correction**

**Background correction method**	**Background velocity (mm/s)**	**RABF per kidney (ml/min)**	**Mean velocity (mm/s)**	**Resistive index**	**Pulsatility index**
None	0 +/− 0	413 +/− 122	253.5 +/− 65.2	0.71 +/− 0.06	1.47 +/− 0.29
Stationary phantom	−2.9 +/− 3.3**	417 +/− 119**	256.5 +/− 64.5**	0.71 +/− 0.06*	1.45 /- 0.29*
Background fit	−4.6 +/− 7.4*	420 +/− 124*	258.1 +/− 68.3*	0.70 +/− 0.06*	1.44 +/− 0.28*

Data are from the initial scanning session (10 subjects × 2 acquisitions (left and right arteries) = 20 acquisitions in total) and are presented as mean +/− SD. (*p < .01, **p < .001 (compared to no background correction)).

**Figure 2:**
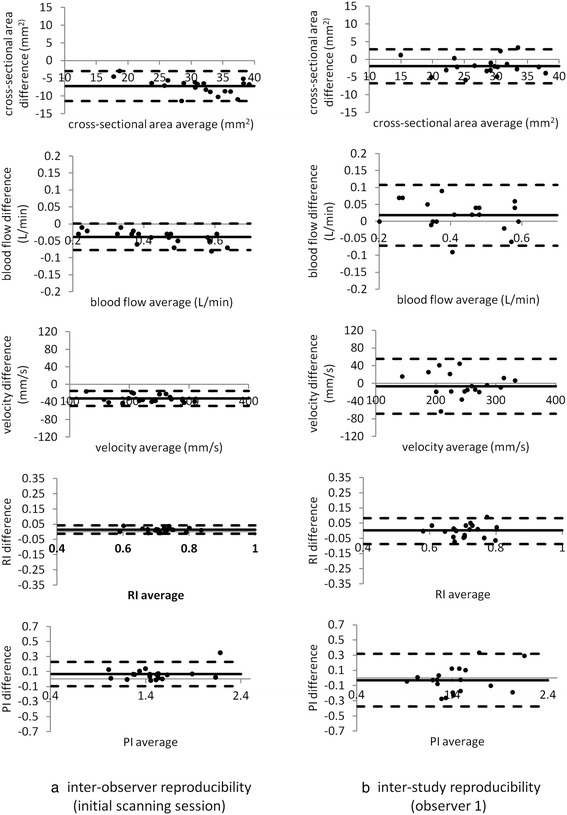
**Bland Altman plots showing inter-observer reproducibility of measurements of cross-sectional area, RABF, mean flow velocity, PI and RI (initial scanning session) (a).** Corresponding plots for the inter-study reproducibility (observer 1) are shown in **(b)**.

**Figure 3:**
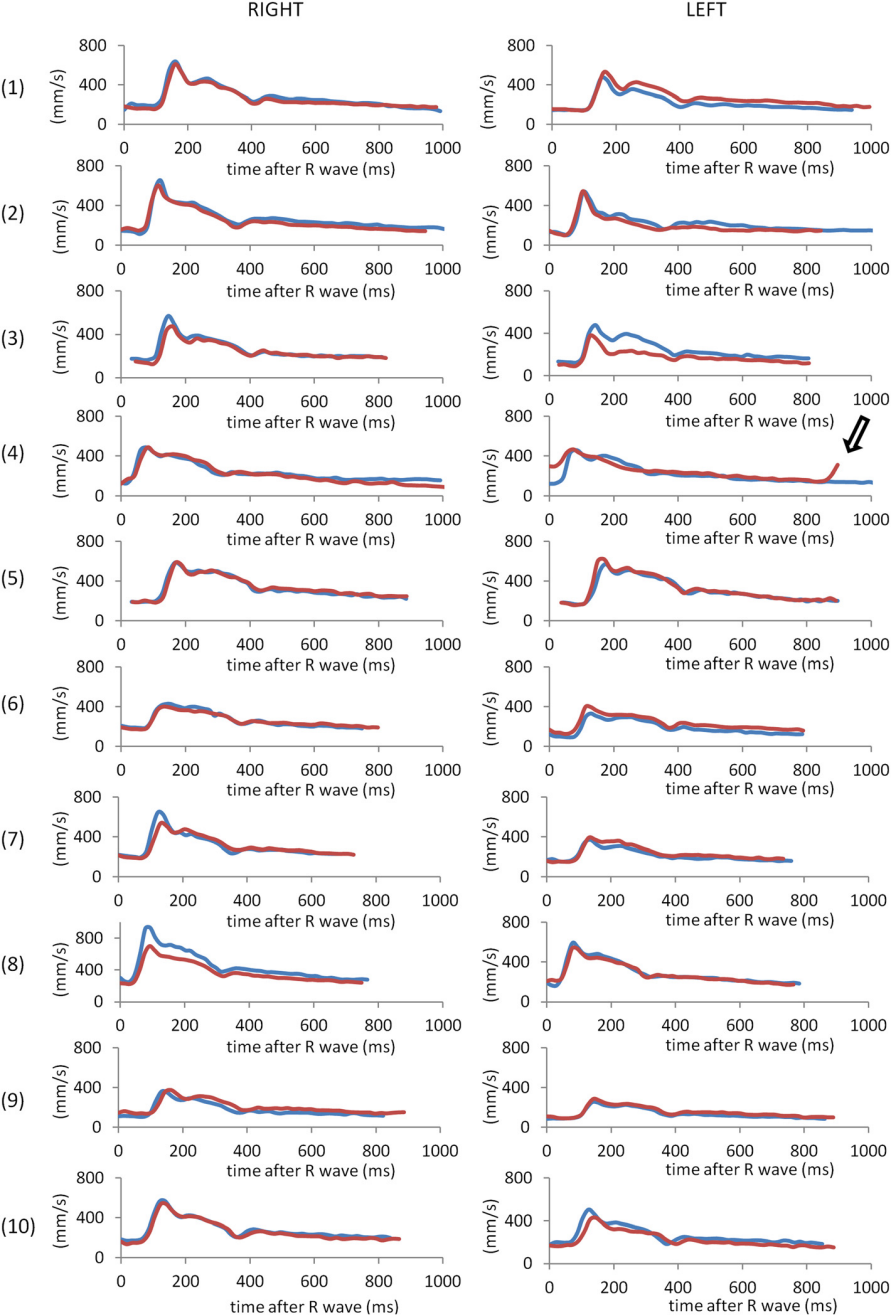
**Velocity-time curves in the initial (blue) and repeat (red) scanning sessions in all 10 subjects, as determined by observer 1.** In the repeat left acquisition in subject 4, a much reduced RR interval (895 ms vs 1095 ms) and ECG mis-triggering (open arrow) resulted in velocity-time curve errors and this acquisition was omitted from all further analyses. (For all graphs, x-axis: time after R-wave (ms), y-axis: mean velocity (mm/s)).

Inter-observer reproducibility (initial scanning session) in cross-sectional area, mean flow velocity, RABF per kidney, RI and PI are presented in the Bland Altman plots of Figure 2(a) and corresponding inter-study reproducibilities (observer 1) are presented in Figure 2(b). The similarity of the temporal flow patterns in the left and right arteries of all 10 subjects in the two scanning sessions (observer 1) is shown in Figure 3. Bland Altman results are summarised in Table 2 while inter-observer and inter-study ICC values and within-subject CVs are reported in Table 3.

**Table 2:** **Inter-observer reproducibility (initial scanning session) and inter-study reproducibility (observers 1 and observer 2) of cross-sectional area, RABF, mean velocity, resistive index (RI) and pulsatility index (PI)**

**Reproducibility**	**Area (mm** ^**2**^ **)**	**RABF per kidney (ml/min)**	**Mean velocity (mm/s)**	**RI**	**PI**
Inter-observer	−7.2+/−2.1**	38.5+/−20.0**	−32.3+/−8.6**	0.01 + −0.01*	0.07+/−0.08*
Inter-study (observer 1)	−2.0+/−2.4	17.9+/−44.8	−6.7+/−31.1	0.00+/−0.04	−0.03+/−0.17
Inter-study (observer 2)	−2.6+/−4.1	24.2+/−59.0	−5.5+/−36.3	0.00+/−0.05	−0.01+/−0.21

(**p < .001, *p < .01).

All data are presented as mean (+/− standard deviation) of the signed differences between measurements.

**Table 3:** **Inter-observer and inter-study Intraclass correlation coefficients (ICCs) and coeffiecients of variation (CV) of cross-sectional area, RABF, mean velocity, resistive index (RI) and pulsatility index (PI)**

		**Area**	**RABF per kidney**	**Mean velocity**	**RI**	**PI**
Inter-observer	ICC	0.73*	0.97*	0.94*	0.97*	0.97*
Inter-study (observer 1)	ICC	0.93*	0.96*	0.93*	0.87*	0.92*
Inter-study (observer 2)	ICC	0.90*	0.95*	0.89*	0.86*	0.93*
Inter-observer	CV (%)	17.1%	7.0%	9.9%	2.0%	4.9%
Inter-study (observer 1)	CV (%)	7.7%	7.9%	8.7%	4.2%	8.4%
Inter-study (observer 2)	CV (%)	9.4%	9.5%	11.4%	4.4%	9.6%

(* = p < .001).

## Discussion

We have developed a high temporal resolution spiral phase velocity mapping sequence which allows robust and reproducible assessment of renal artery haemodynamics at 3 Tesla. In addition, for the first time using an MR method, we report parameters of renal artery blood flow pulsatility and their reproducibility. An analysis of background phase errors has also been performed and we have shown that these amount to <2% of measured velocities throughout the cardiac cycle and can therefore be ignored.

Spiral k-space coverage is highly efficient and this can be used to acquire higher temporal resolution data in shorter acquisition periods than is possible using conventional segmented Cartesian acquisitions. Spiral phase velocity mapping of renal blood flow per minute has previously been validated against the technique of p-aminohippuric acid clearance haematocrit [21]. However, the temporal resolution in this study was much poorer than ours (55 ms compared to 19 ms), precluding accurate determination of temporal flow details and the assessment of pulsatility indices. The breath-hold duration was also longer (20 cardiac cycles compared to 17 cardiac cycles).

Values of RI (0.71 +/− 0.06) and PI (1.47 +/− 0.29) in this healthy subject cohort are similar to, but slightly higher than, values obtained from Doppler studies (RI: 0.67 (range 0.64 - 0.70), PI: 1.19 (range 0.93 – 1.25)) [33]. In Doppler ultrasound studies, measurement of RI is generally performed at the interlobar or arcuate renal artery level while in our study, assessment is performed in the proximal vessels where peak velocity is higher and end-diastolic velocity is lower, resulting in increased RI and PI [34]. The total RABF (left + right) was 826 +/− 204 ml/min which is similar to values found by Bax et al. (838 +/− 244 ml/min in 40 healthy volunteers) [23] and by Dambreville et al. (1130 +/− 88 ml/min in 6 healthy volunteers (repeated studies)) [26] and which is consistent with an RABF per kidney of 365 +/− 119 ml/min reported by Khatir et al. (9 healthy volunteers) [24].

Background phase can be an important source of error in MR phase velocity mapping and we have minimised this by careful positioning of the region of interest close to isocentre [35]. Correction is generally performed by fitting a plane to user defined stationary points within the image. However, the accuracy of this technique relies on there being sufficient stationary material around the region of interest to enable accurate fitting. For the renal arteries, the inter-vertebral disks are always seen to one side of the vessel of interest (Figure 1) and have previously been used as reference points for renal blood flow assessment [27]. Finding stationary material close to the other side however is difficult and can compromise the accuracy of the fit. Using a stationary phantom dataset with identical acquisition parameters and image plane orientation to the *in vivo* dataset is the most accurate method of determining background values [31] although differences between the patient and the phantom may mean that the correction is imperfect. In addition, this method is cumbersome and time-consuming and for these reasons, it is difficult to implement in clinical practice. We have shown that for the renal arteries, the background velocity corrections are approximately 1 - 2% of the mean velocity through the cardiac cycle (−2.9 +/− 3.3 mm/s for the stationary phantom method and −4.6 +/− 7.4 mm/s for the background fit method). The greater standard deviation for the background fit correction is likely to reflect reduced accuracy due to the lack of nearby stationary tissue and the inherent subjectivity in defining the stationary points. In practice, although the correction results in statistically significant differences in flow parameters, as shown in Table 1, these differences are so small that they are clinically unimportant and background velocity compensation can be ignored.

Analysis of the inter-observer reproducibility of flow parameters showed statistically significant differences between observers (Table 2, Figure 2). These derived primarily from systematic differences in defining the renal cross-sectional area, with the average difference between the two observers being 23% of the mean value. These differences are not wholly surprising as, while both observers were highly experienced in CMR and used fixed window display levels and widths when drawing the ROIs, the vessel diameters are small and the pixel size is relatively large (1.4 mm × 1.4 mm, reconstructed to 0.7 mm × 0.7 mm). The correlation between the two area measurements however was high with a Pearson correlation coefficient of 0.95 (confirming that this is a systematic bias rather than a random variation) and an ICC of 0.73. The standard deviation of the differences divided by the mean is therefore relatively small: area 7%, flow 5%, mean velocity 4%, PI 5%, RI 2%. In this study, cross-sectional area regions of interest were manually drawn on the diastolic segmented gradient echo scout images rather than on the spiral images as these had higher spatial resolution (1 mm × 1 mm compared to 1.4 mm × 1.4 mm) and were not subject to off-resonance or motion blurring. Changes in breath-hold position between this scout acquisition and the spiral flow acquisition may lead to inaccuracies although these were not apparent from visual inspection of the images. In addition, while the window level and width were defined in a reproducible way for each vessel, the definition of the border is still somewhat subjective and an automatic or semi-automatic method of region of interest generation based on intensity contours would be beneficial and would potentially reduce inter-observer differences. This could also potentially enable the region to be defined on every individual cine frame and therefore take into account changes in cross-sectional area through the cardiac cycle. The accuracy of the region of interest definition could also be improved by increasing the spatial resolution and the number of pixels across the vessel diameter [36,37].

There are numerous statistical methods of assessing reproducibility and meaningful comparisons between studies are complicated by the fact that reproducibility data is reported in different ways in different studies and indeed, it is not always evident from the text exactly how calculations have been made. In this study, we have used Bland Altman analysis (Table 2, Figure 2), the intraclass correlation coefficient (ICC) (Table 3) and the within-subject coefficient of variation (Table 3). Inter-study reproducibility of flow parameters was similar for both observers (Table 2). For pulsatility measures, the within-subject CVs are low, being 4.2% and 4.4% for RI (observers 1 and 2 respectively) and 8.4% and 8.8% for PI (observers 1 and 2 respectively) (Table 3). There are no MR reproducibility studies for renal RI and PI with which to compare these figures. Doppler studies, however, have reported CVs of 4.8% (RI) and 6.8% (PI) [38] and of 6% (RI) and 9% (PI) [39] which agree well with values obtained in this current study. The lower within-subject CV of RI compared to PI is such that it is RI that has generally been used to investigate disease state [38]. In addition, other studies have reported an RI reproducibility coefficient (defined as twice the standard deviation of the paired differences between repeat studies) of 0.080 and 0.060 for two observers in 100 healthy volunteers [40], and 0.074 and 0.086 for two observers in 18 renal allograft recipients [41]. These values are comparable to the reproducibility coefficients obtained in this study (0.086 and 0.091 for observers 1 and 2 respectively). For RABF assessment, the within-subject CVs in this study were 7.9% (observer 1) and 9.5% (observer 2). These compare favourably with values in previously published MR studies of 8.1% [26], 23% [23] and 8.3% [24]. The inter-study ICCs for flow, mean velocity, RI and PI were all >0.85 for both observers (Table 3) which suggests that <15% of differences between studies is due to errors in the measurement process. ICC values above 0.8 are regarded as representing good – high reliability [42].

Assessment of RI and PI requires accurate determination of the sharp systolic velocity peak which in turn requires high temporal resolution. In this study, the acquired temporal resolution is 19 ms which is approximately 2–3 times higher than that of previous studies [21,26]. Down-sampling the velocity time curves presented in Figure 3 results in the systolic peak velocity changing from 509 +/− 161 mm/s to 485 +/− 155 mm/s (with down-sampling factor 2) to 477 +/− 158 mm/s (with down-sampling factor 3). This results in the RI and PI values falling from 1.47 +/− 0.29 to 1.36 +/− 0.24 (−7% with down-sampling factor 2) and to 1.28 +/− 0.27 (−13% with down-sampling factor 3). The corresponding PI values fall from 0.71 +/− 0.06 to 0.69 +/− 0.05 (−3%) and to 0.67 +/− 0.07 (− 6%) respectively. The reproducibility of the peak velocity, RI and PI (expressed as the standard deviation of the signed differences as a percentage of the mean) is also degraded by down-sampling the curves by factors of 2 and 3 (from 14% to 16% to 19% (peak velocity), 6% to 7% to 10% (RI) and 12% to 14% to to 21% (PI)). In practice, the effects of reduced temporal resolution would be greater than presented here as reduced temporal resolution results not only in sampling at different time-points in the cardiac cycle (as in this simple simulation) but also in a much larger acquisition window.

The limitations of this study include the absence of a comparison of our technique for measuring pulsatility and RABF with any of the others currently employed. However, it should be noted that there is no widely accepted gold-standard technique suitable to use in normal human subjects [43]. In addition, as discussed above, the low spatial resolution of the cross-sectional images impacts on the accuracy of the region of interest definition and the manual delineation of the vessel cross-sectional areas has resulted in a bias between observers. The use of a fixed cross-sectional area throughout the cardiac cycle is also a limitation of our technique. The study cohort is also small and consists of only healthy volunteers. In addition, our volunteers were relatively young and we have not attempted to investigate the relationships between pulsatility indices and age [44,45].

## Conclusions

In conclusion, we have developed a high temporal resolution spiral phase velocity mapping technique for the rapid and reproducible assessment of the temporal patterns of renal artery blood flow and the reproducible assessment of the resistive and pulsatility indices at 3 Tesla. The technique is clinically relevant and provides an excellent research tool for assessment of renal haemodynamics in cardiovascular research.

## Abbreviations

TR: Repeat time
RI: Resistive index
PI: Pulsatility index
RABF: Renal artery blood flow
RSD: Renal sympathetic denervation
TE: Echo time
PSV: Peak systolic velocity
MDV: Minimum diastolic velocity
MV: Mean velocity
ICC: Intraclass correlation coefficient
